# Lysyl Oxidase and the Tumor Microenvironment

**DOI:** 10.3390/ijms18010062

**Published:** 2016-12-29

**Authors:** Tong-Hong Wang, Shih-Min Hsia, Tzong-Ming Shieh

**Affiliations:** 1 Tissue Bank, Chang Gung Memorial Hospital, Taoyuan 33305, Taiwan; cellww@gmail.com; 2 Research Center for Industry of Human Ecology, Chang Gung University of Science and Technology, Taoyuan 33303, Taiwan; 3 Graduate Institute of Health Industry Technology, Chang Gung University of Science and Technology, Taoyuan 33305, Taiwan; 4 Liver Research Center, Chang Gung Memorial Hospital, Taoyuan 33303, Taiwan; 5 School of Nutrition and Health Sciences, College of Nutrition, Taipei Medical University, Taipei 11031, Taiwan; 6 Nutrition Research Center, Taipei Medical University Hospital, Taipei 11031, Taiwan; 7 Department of Dental Hygiene, College of Health Care, China Medical University, Taichung 40402, Taiwan

**Keywords:** lysyl oxidase, microenvironment, tumor progression, tumor suppressor, metastasis

## Abstract

The lysyl oxidase (LOX) family of oxidases contains a group of extracellular copper-dependent enzymes that catalyze the cross-linking of collagen and elastin by oxidation, thus maintaining the rigidity and structural stability of the extracellular matrix (ECM). Aberrant expression or activation of LOX alters the cellular microenvironment, leading to many diseases, including atherosclerosis, tissue fibrosis, and cancer. Recently, a number of studies have shown that LOX is overexpressed in most cancers and that it is involved in the regulation of tumor progression and metastasis. In contrast, a few reports have also indicated the tumor-suppressing role of LOX. In this short review, we discuss recent research on the correlations between LOX and cancer. Further, the role of LOX in tumor microenvironment remodeling, tumorigenesis, and metastasis and the underlying mechanisms have also been elucidated.

## 1. Introduction

The lysyl oxidase (LOX) family includes five members—LOX and LOX-like (LOXL) 1, 2, 3, and 4. All of them have similar catalytic activity owing to the highly conserved C-terminal region containing the copper binding domain, residues for lysine tryosylquinone (LTQ), cofactor formation, and a cytokine receptor-like (CRL) domain. The N-terminal region of LOX and LOXL1 are quite different from those of LOXL 2–4. LOX and LOXL1 contain prodomains, while LOXL 2–4 contain four scavenger-receptor cysteine-rich domains each. The mature form of LOX and LOXL1 need extracellular processing, but LOXL 2–4 do not. The LOX mRNA is translated to pre-pro-protein (pre-pro-LOX, 48 kDa), followed by the incorporation of copper, cleavage of 21 amino acids, glycosylation of the N-terminal, and tertiary folding, to form the inactive LOX proprotein (pro-LOX, 50 kDa) in the cytoplasm. The N-terminal of pro-LOX is then secreted out of the cell and cleaved by procollagen-C-proteinase (BMP-1) to become LOX propeptide (LOX-PP, 18 kDa) and mature active LOX (32 kDa). Extracellular LOX and LOX-PP can then re-enter cells from the extracellular environment to exert their biological activities.

LOX is a secreted copper-dependent amine oxidase, which is expressed in various cell types such as basal and suprabasal keratinocytes, fibroblasts, adipocytes, osteoblasts, smooth muscle cells, and endothelial cells. The most well-known function of LOX is the initiation of the crosslinking of collagens and elastin [1,2]. Such modifications of structural components of the extracellular matrix (ECM) stabilize fibrous deposits and contribute to tissue strength and integrity in the connective tissue. Bone and cartilage are types of connective tissues in the body and LOX, also playing an important role in bone formation. Inhibition of *LOX* gene expression results in decreased osteoblastic differentiation [3]. In addition, LOX can also activate the promoters of collagen III and elastin [4,5], but its indirect involvement represses the promoter of cyclin D1 [6]. Thus, LOX plays an important role in the ECM, both intra- and extra-cellularly; in the dermis during normal physical development, aging, wound repair; and in fibrotic disorders including liver cirrhosis and atherosclerosis [7]. LOX mRNA or protein are overexpressed in various organs such as the skin [8], oral submucosa [9], liver [10,11], lung [12], kidney [13], and bone marrow [14] during fibrosis. In epigenetics, LOX is known to interact with the lysine-rich N-terminal tail of histones H1 and H2, but the mechanisms and effects have not been clarified so far [15].

**Figure 1:**
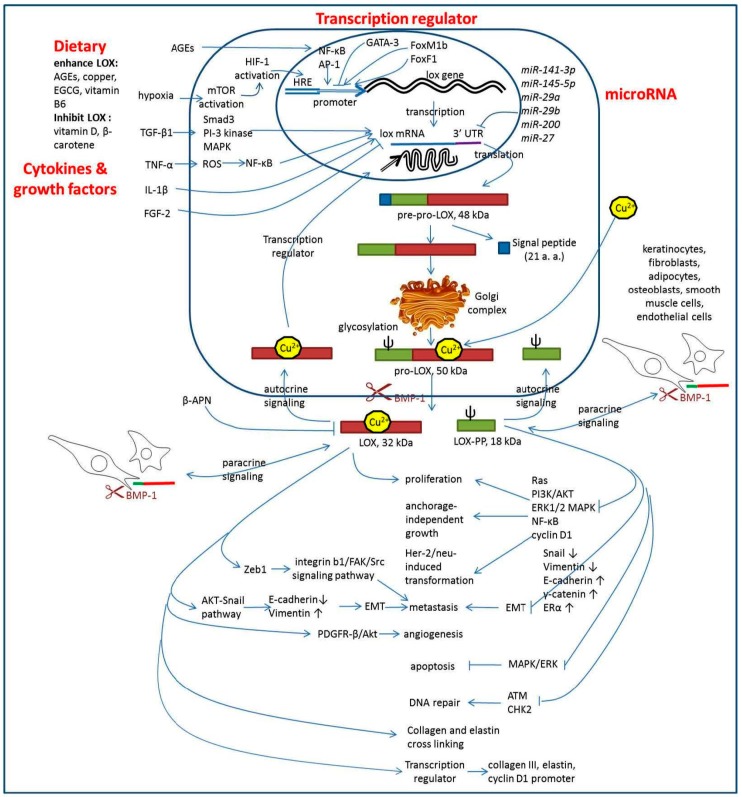
Regulation and signaling of LOX in the tumor microenvironment. LOX was regulated by cytokines and growth factors, dietary, transcription regulators, and microRNAs. The up arrows, up-regulations; the down arrows, down-regulations.

LOX also plays a role in cancer by enhancing cancer cell proliferation, invasion, metastasis, and angiogenesis [16,17,18,19,20]. However, LOX has also been shown to have tumor suppressor function [21], and the more recent studies have shown that the tumor suppressor function is due to LOX-PP [22,23,24,25]. The mature active LOX and LOX-PP play opposite roles in cancer progression. However, whether LOX enhances or suppresses tumor progression in various tissues, sites, sizes, and stages of tumors is still controversial [26]. This review is focused on the expression, functions, upstream regulator, and downstream molecules of LOX in tumors. The effect of dietary components on LOX activity and the possibility of using LOX as a tumor therapy target have also been discussed. The simplified regulation and signaling of LOX in the tumor microenvironment was summarized in Figure 1.

## 2. LOX Enhances Tumor Progression

LOX is overexpressed in various tumors such as oral cancer, tumor endothelial cells [20], gastric cancer [27], breast cancer [28], and anaplastic thyroid cancer (ATC) [29]. LOX overexpression enhances cell proliferation and tumor angiogenesis in oral squamous cell carcinoma (OSCC) [19], colorectal cancer (CRC) [30], and astrocytomas [31]. During tumor development, tumor cells constantly communicate with the surrounding microenvironment to induce tumor cell proliferation, epithelial-to-mesenchymal transition (EMT), migration, invasion, angiogenesis, and metastasis. Invasion and metastasis are the main steps in determining tumor malignancy. However, in addition to the various genetic aberrations accumulating in the process of tumor progression, the microenvironment of the tumor cells and resident nonmalignant cells is also important [18]. In this process of epithelial cells lose their cell–cell adhesion and polarity, and gain migrate and invasive abilities to become mesenchymal stem cells was called EMT. Epithelial cells express high levels of *E*-cadherin, whereas mesenchymal cells express high levels of *N*-cadherin, vimentin, and fibronectin. Invasion is the first step before metastasis, which is enabled by EMT. LOX downregulation significantly increases the E-cadherin level and decreases the vimentin level [17,32], which shows that LOX can cause cancer cells to favor metastatic spread, which has been demonstrated in both in vitro and in vivo experiments. High LOX expression induces metastasis in various cancers such as OSCC [33], CRC [30], breast cancer [34], ATC [29], and lung adenocarcinoma [35]. When the tumor size becomes more than 1–2 mm^3^, the central tumor cells are subjected to a hypoxic microenvironment; this induces the activation of the hypoxia-inducible factor (HIF) to facilitate its binding to the hypoxia-response element (HRE) to induce cell proliferation and expression of angiogenesis factors such as epidermal growth factor (EGF) and vascular endothelial growth factor (VEGF). The HRE of the *LOX* promoter is upregulated by HIF in breast and head and neck cancers [36,37]. Further, the combination of HIF, LOX, and VEGF not only enhances tumor cell proliferation, but also induces angiogenesis to induce the cells to serve a metastasis path. Moreover, it has been shown that the LOX inhibitor, β-aminopropionitrile (β-APN), decreases the hypoxia-induced invasion and migration of cervical cancer cells [38]. Additionally, cancer-stromal cell interactions mediated by HIF also promote angiogenesis, lymphangiogenesis, and metastasis [39]. Lysyl oxidase secreted by tumor endothelial cells promotes angiogenesis and metastasis in colorectal cancer and breast cancer [20,40]. Taken together, these data indicate that, irrespective of whether LOX is secreted from the tumor cell, surrounding fibroblasts in the connective tissue, or the endothelial cells of nearby vessels, LOX enhances tumor progression in the tumor cell microenvironment.

## 3. *LOX* as a Tumor Suppressor Gene

Despite the fact that enough evidence indicates that LOX enhances tumor progression, several studies have also showed that *LOX* acts as a tumor suppressor gene. LOX mRNA and protein expression is lower in tumor tissues with lymph node metastasis and deep muscular layer infiltration in upper digestive tract carcinomas [41], human osteosarcoma tissues [42], and gastric cancers [21]. In 1990, Contente S was the first to show that the *LOX* gene can reverse Ha-ras (v-Ha-ras Harvey rat sarcoma viral oncogene homolog) induced transformation of both NIH 3T3 fibroblasts and cloned rat embryo fibroblasts; therefore, LOX has also been named as the ras recision gene (*rrg*) [43,44]. The transformed phenotype of ras-expressing embryonic fibroblasts with a null mutation in the transcriptional activator IFN regulatory factor 1 (IRF-1) allele can be suppressed by the expression of the LOX cDNA [45]. Furthermore, transformation of cells with ras causes the methylation of the *LOX* promoter, which leads to the transcriptional suppression of LOX [46]. In another study, *LOX* was found to be inactivated by methylation and loss of heterozygosity in human gastric cancers [21]. While the enzymatic activity and transcriptional activating properties belong to the 32-kDa LOX protein, the tumor suppressing ability of the *LOX* gene is from the 18-kDa LOX-PP. LOX-PP can be taken up by cells and is therefore an autocrine and paracrine molecule. The cellular uptake of LOX-PP occurs via the PI3K-dependent macropinocytosis pathways and dynamin- and caveola-dependent pathway [23]. Extracellular LOX-PP inhibits the PI3K/AKT and ERK1/2 MAP kinase signaling pathways and lowers the downstream nuclear factor kappa-light-chain-enhancer of activated B cell (NF-κB) and cyclin D1 levels to inhibit tumor proliferation and anchorage-independent growth [22,47]. Upstream of RAS, LOX-PP inhibits Her-2/neu-driven breast cancer cell transformation by suppressing Akt and NF-κB. LOX-PP can also reverse Her-2/neu-induced EMT, as indicated by reduced levels of Snail and vimentin and upregulation of E-cadherin, γ-catenin, and estrogen receptor α (ERα) [48]. LOX-PP also inhibits radiation-induced activating phosphorylation of ataxia-telangiectasia mutated (ATM) and checkpoint kinase 2 (CHK2) to inhibit DNA repair pathways in prostate cancer xenograft growth [24]. LOX-PP expression can also induce apoptosis, probably through the downregulation of the MAPK/ERK pathway, and is associated with clinical tumor stage and distant metastasis in hepatocellular carcinoma [25]. Overall, the anticancer activities of LOX-PP have been proved to inhibit cell transformation, DNA repair, proliferation, EMT, and anchorage-independent growth, but induce apoptosis in various tumor cells.

## 4. LOX Regulation

From the above data, it is clear that it is extremely important to maintain proper intracellular LOX expression and activation. Aberrant expression or activation of LOX often leads to many diseases including tissue fibrosis and cancer [49,50]. LOX expression can be regulated at the transcriptional level, polypeptide modification level, enzyme activity levels, or at the protein distribution level. Cytokines and growth factors such as transforming growth factor-β1 (TGF-β1), tumor necrosis factor-α (TNF-α), interleukin-1β (IL-1β), and fibroblast growth factor-2 (FGF-2) have also been found to be involved in the regulation of LOX expression. Among these, TGF-β, one of the key cytokines involved in regulating the ECM, promotes LOX mRNA expression via the activation of Smad3, PI-3 kinase, and mitogen-activated protein kinase (MAPK) signaling [51,52,53]. TGF-β increases steady state LOX mRNA level in a dose- and time-dependent manner, thus regulating the structure and stability of the ECM in cardiac fibroblasts responsible for cardiac fibrosis and cardiac dysfunction [53]. Chronic inflammation is also considered a key driver of LOX expression. TNF-α, a pro-inflammatory cytokine, induces LOX expression via the reactive oxygen species-activated NF-κB/extracellular signal-related kinase pathway, thus promoting the progression of cardiac fibrosis and breast cancer metastasis [54]. However, in endothelial cells, TNF-α downregulates LOX mRNA expression and enzymatic activity in a dose- and time-dependent manner, and this effect associated with an impairment of endothelial barrier function [55]. Thus, it can be seen that the regulation of LOX expression by TNF-α appears to depend on tissue type. Numerous reports have shown that IL-1β, another important chemical mediator in acute inflammation, also upregulates LOX expression in adult skin and lung fibroblasts and thus attenuates myofibroblast formation and ECM production [56,57,58]. In addition, the autocrine growth factors, basic bFGF and FGF-2, have been reported to downregulate LOX transcription in tumorigenic-transformed RS485 cells, and this effect might be associated with cell transformation [59].

In addition, several transcription factors also regulate LOX expression. GATA-binding protein 3 (GATA-3), a transcription factor essential for normal mammary gland development and luminal cell differentiation, suppresses *LOX* gene expression by regulating the methylation of the *LOX* gene promoter [60]. FoxM1b, a transcription factor often overexpressed in human cancers, enhances LOX and LOXL2 expression by directly binding to the *LOX* and *LOXL2* gene promoters, further activating the downstream AKT-Snail pathway and promoting EMT and hepatocellular carcinoma metastasis [61]. Hypoxia-inducible factor 1α (HIF-1α), a key transcription factor induced in hypoxic cells, also has been identified to be involved in regulating LOX expression [39]. Moreover, hypoxia has been shown to be a microenvironmental factor in many diseases and induces tumor expression of LOX through HIF-1α to enhance cell–matrix adhesion, migration, invasion, and metastasis [62]. In addition, hypoxia also induces stromal expression of LOX, which causes collagen linearization, increases ECM stiffness, and induces epithelial phenotype loss in cancer cells, thus enhancing tumor cell invasion through ECM remodeling [63]. Some HIF-1α upstream regulatory molecules such as NOTCH, mTOR (mechanistic target of rapamycin) and LKB1 can also regulate LOX expression indirectly through HIF-1α and mediates cancer progression [64,65,66,67,68,69]. In lung cancer, Zeb1 promotes invasion and metastasis by activating the integrin β1/FAK/Src signaling pathway by inducing the expression of LOX and LOXL2 [70]. Moreover, studies in breast cancer reveal that Forkhead Box F1 (FoxF1) promotes cancer cell migration by upregulating LOX and suppressing Smad2/3 signaling, whereas nuclear factor I-C2 (NFI-C2) suppresses EMT, motility, invasiveness, and tumor growth of breast cancer by downregulating *LOX* gene expression [71].

Numerous micro-RNAs have also been shown to be involved in the regulation of LOX expression. In clear cell renal cell carcinoma, both miR-141-3p and miR-145-5p directly bind to the 3′ untranslated region of the LOX mRNA and cooperatively downregulate LOX expression, thereby suppressing the progression of clear cell renal cell carcinoma [72]. In granulosa lutein cells, miR-29a directly downregulates LOX expression; as a result, TGF-β1 upregulates LOX expression by downregulating miR-29a [73]. In the same way, miR-29b inhibits collagen maturation in hepatic stellate cells by downregulating LOX expression [74]. In thyroid cancer, LOX has been identified as the direct target of miR-30a, and downregulation of miR-30a mediates the upregulation of LOX and progression of thyroid cancer [29]. miR-200b directly inhibited LOX expression, leading to decreased invasion in a mouse breast tumor model [75]. This miR-200 was then downregulated to increase LOX expression in cancer-associated fibroblast to trigger tumor cell invasion [76]. miR27 represses the final differentiation of mesenchymal stem cells to the adipogenic lineage by targeting LOX [77]. In addition to the above-mentioned miRs that directly target the *LOX* gene, there are many other miRs that regulate LOX expression by targeting the cytokines, growth factors, and transcription factors that indirectly affect LOX expression.

## 5. Downstream Signaling of LOX

Extracellular LOX is composed of enzymatic LOX and LOX-PP; however, the downstream signaling of LOX has only been addressed in a few reports so far. LOX-PP expression reduces cell proliferation, cell migration, anchorage-independent growth, and tumorigenesis in immunodeficient mice. As described above, LOX-PP inhibits the ERK/MAPK pathway and many other pathways involved in cell cycle progression during Ewing’s sarcoma [78] and has antitumoral properties. In contrast, the C-terminal domain of LOX, which contains the enzymatic activity, has the opposite effects. LOX regulates cell adhesion and migration by inducing the activation of SRC kinase and focal adhesion kinase (FAK) and further activates downstream signaling cascades [70,79,80,81]. LOX can also promote the progression of colorectal cancer by activating the phosphoinositide 3-kinase-Akt signaling pathway [82]. Furthermore, by activating platelet-derived growth factor receptor β (PDGFR-β)/Akt signaling, LOX can promote angiogenesis by enhancing vascular endothelial growth factor expression and secretion [40]. These results corroborate that the tumor suppressor activity of LOX is mediated exclusively by its propeptide domain. Thus, the proteolytic removal of LOX pro-peptide by BMP-1/Tolloid metalloproteinases after secretion is essential to the exhibition of its oxidase activity and protumor properties.

## 6. Dietary Components Regulate LOX Activity

In previous studies, many dietary components or herbal extracts such as advanced glycation end products (AGEs), copper, green tea, vitamin D, β-carotene, vitamin B6, *Calendula officinalis* have been shown to regulate LOX activity [83,84,85,86,87,88,89,90,91]. AGEs are a group of reactive compounds formed by the Maillard reaction. They can also be formed during the oxidation of lipids or nucleotides and nonenzymatic glycation. AGEs are involved in the pathogenesis of diabetic complications including cardiovascular diseases, obesity, retinopathy, nephropathy, neuropathy, vascular complications, and cancer [92,93,94,95]. AGE modification of target proteins from the ECM induces crosslinking, which is often associated with thickening of the basement membrane. AGEs trigger NF-κΒ- and AP-1-mediated upregulation of LOX and ET-1 via the AGE/RAGE/MAPK signaling cascade in human endothelial cells [91]. High levels of AGEs have also been shown to stimulate LOX activity and subsequent collagen deposition in ovarian tissues of patients with polycystic ovary syndrome (PCOS) [96]. Moreover, AGEs can potentially regulate the expression of the *LOX* gene, which leads to increased LOX expression that is associated with distorted endothelial homeostasis. LOX, which is over-expressed in the cardiovascular system in obesity, emerges as a potential mediator of cardiovascular remodeling as well [91]. LOX deficiency induces type V of Ehlers–Danlos Syndrome (EDS), which is caused by a defect in the structure, production, or processing of collagen or proteins that interact with collagen [97]. Menkes syndrome, which is an X-linked recessive disorder that affects copper levels in the body, leading to copper deficiency and low LOX activity [98], has also been proposed to be a form of EDS. The herbal extract of *Calendula officinalis* can also inhibit LOX mRNA expression in B16F-10 melanoma-bearing animals [99]. Increased LOX expression suffices to induce collagen accumulation and fibrosis in vivo [100]. An increase in myocardial LOX expression has also been reported in animal models of metabolic syndrome [101]. Leptin induces LOX expression in cardiac myofibroblasts and vascular smooth muscle cells (VSMCs) [102], and serum leptin levels have been found to correlate with cardiovascular disease risk and metabolic syndrome.

## 7. LOX as a Target for Anti-Cancer Therapy

Because LOX has been associated with aggressive cancers and metastasis, it is important to characterize the intracellular functions of LOX. LOX can be a molecular target for anti-cancer treatment [103], as it has been reported to increase migration, invasion, and metastasis dissemination through its capacity to regulate collagen cross-linking and ECM stiffening in different kinds of cancer [49,50]. This activity has also been implicated recently in senescence escape and cooperation with oncogenic signals to promote pancreatic ductal adenocarcinoma (PDAC) formation in mice. Moreover, Benjamin et al. have found that the activities of members of the LOX family are both a novel target to improve the effect of chemotherapy as well as a novel biomarker to predict gemcitabine benefits in PDAC. Furthermore, it is also possible that targeting the activities of members of the LOX family will improve the efficacy of chemotherapies against different kinds of solid tumors [104]. Inhibition of LOX expression has been shown to improve drug diffusion and increase the efficacy of cytotoxic treatment in 3D tumor models [105]. Schutze et al. also showed that LOX activity modulates the physical barrier function of the ECM for small molecule drugs, thus influencing their therapeutic efficacy [105]. Therefore, targeting this process has the potential to significantly enhance therapeutic efficacy in the treatment of malignant diseases.

Hypoxia is a powerful and independent prognostic indicator of poor clinical outcome for patients with cervical and other types of cancers. Hypoxia can enhance tumor invasiveness, metastases, and resistance to chemotherapy [39]. In a previous study, Yang et al. found that LOX protein expression and catalytic activity were upregulated in cervical cancer cells following exposure to hypoxia [38]. In this cervical cancer study, exposure to hypoxia conferred a mesenchymal-like morphology to the HeLa and SiHa cells, which is consistent with the upregulation of α-smooth muscle actin (α-SMA) and vimentin and downregulation of E-cadherin [38]. In cervical cancer study, β-aminopropionitrile (βAPN) inhibit LOX expression will blocked the EMT phenomenon of cervical cancer cells and inhibited invasion and migration under hypoxia in vitro; these data provide insights into the therapy and prevention of cervical cancer metastasis [38]. The LOX inhibitor, βAPN, abolished metastasis, thus offering preclinical validation of this enzyme as a therapeutic target [34,37]. In another study, Kanapathipillai et al. showed that nanoparticles coated with a LOX inhibitory antibody bind to ECM and suppress mammary cancer cell growth and invasion in vitro [106]. Further, as discussed above, miRNA can also suppress LOX expression. Boufraqech et al. showed that miR30a inhibits LOX expression and anaplastic thyroid cancer progression [29]. LOX expression has also been found to be related to radioresistance. For example, Gong et al. showed that non-small cell lung cancer cells with high LOX expression showed hypoxia-induced radioresistance [107]. Therefore, using LOX as a target for anti-cancer drug discovery has great potential for cancer therapy in the future.

## 8. Conclusions

In conclusion, the current review indicates that LOX can crosslink collagen and elastin by oxidation, thus maintaining the rigidity and structural stability of ECM. The ECM plays an important role in the tumor microenvironment for invasiveness and metastases. Therefore, the development of drugs targeting LOX may have therapeutic significance for the prevention and treatment of tumor metastasis.
